# Surgical Treatment of Pediatric Epileptic Encephalopathies

**DOI:** 10.1155/2013/720841

**Published:** 2013-10-30

**Authors:** J. Fridley, G. Reddy, D. Curry, S. Agadi

**Affiliations:** ^1^Department of Neurosurgery, Baylor College of Medicine, 1709 Dryden, Houston, TX 77030, USA; ^2^Department of Surgery, Section of Pediatric Neurosurgery, Texas Children's Hospital, CCC Suite 1230, 6621 Fannin Street, Houston, TX 77030, USA; ^3^Department of Neurology, Baylor College of Medicine, 6501 Fannin Street, NB302, Houston, TX 77030, USA; ^4^Department of Pediatrics, Section of Neurology and Developmental Neuroscience, One Baylor Plaza, Houston, TX 77030, USA

## Abstract

Pediatric epileptiform encephalopathies are a group of neurologically devastating disorders related to uncontrolled ictal and interictal epileptic activity, with a poor prognosis. Despite the number of pharmacological options for treatment of epilepsy, many of these patients are drug resistant. For these patients with uncontrolled epilepsy, motor and/or neuropsychological deterioration is common. To prevent these secondary consequences, surgery is often considered as either a curative or a palliative option. Magnetic resonance imaging to look for epileptic lesions that may be surgically treated is an essential part of the workup for these patients. Many surgical procedures for the treatment of epileptiform encephalopathies have been reported in the literature. In this paper the evidence for these procedures for the treatment of pediatric epileptiform encephalopathies is reviewed.

## 1. Introduction

 Pediatric epileptic encephalopathies are a group of epileptiform disorders in which the epileptic processes themselves are believed to contribute to disturbances in neurologic function [1]. When this term was initially introduced, only a few conditions were included in this group: early myoclonic encephalopathy (EME)/Ohtahara syndrome, West syndrome, myoclonic epilepsy in infancy, Dravet syndrome, myoclonic status in nonprogressive epilepsy (MSNE), epilepsy with myoclonic astatic seizures (MAE), Lennox-Gastaut syndrome (LGS), and epileptic encephalopathy with continuous spike and wave during sleep (CSWS) including Landau-Kleffner syndrome [2]. In 2010 the International League against Epilepsy (ILAE) redefined this condition to include any epilepsy that can cause encephalopathy. In addition, focal or lesional epilepsy, both under-treated and particularly resistant to treatment, can also lead to global disturbance of brain function. Unfortunately many patients with these conditions are considered to have drug-resistant epilepsy (DRE), defined as failure of two tolerated, appropriately chosen antiepileptic medications [3]. Surgery, though uncommonly performed in patients with pediatric encephalopathy, can be a treatment option in carefully selected DRE patients [4, 5]. Surgical options include vagus nerve stimulation (VNS), corpus callosotomy (CC), lesionectomy, lobectomy, hemispherotomy/hemispherectomy, stereotactic thermal ablation, multiple subpial transection (MST), and deep brain stimulation (DBS). 

## 2. Surgical Options in Epilepsy Management

Placement of a VNS involves wrapping electrode leads around the left vagus nerve in the neck and connecting the electrode wire to a subcutaneous battery. Electrical stimulation is propagated along vagus nerve afferents to the cerebrum. The exact mechanism of action in the brain due to VNS is unclear, although there are multiple proposed theories [6]. VNS is a FDA treatment for DRE. This is in part due to the results of two large randomized-controlled trials in adolescents and adults that examined the efficacy of VNS versus a sham procedure [7, 8]. Both studies found a significant reduction in seizure frequency with VNS. A recent meta-analysis of VNS efficacy found that VNS may be effective in children as well [9]. In fact, VNS has been reported in patients less than 3 years old, blurring the age boundary at which this procedure may reasonably be performed [10].

CC is a procedure that involves performing a craniotomy, and surgically dividing the corpus callosum to prevent seizure travel between hemispheres. The entire corpus callosum versus only a partial division may be performed. Complications unique to CC include possible akinesia, mutism, hemiparesis, decreased speech, disconnection syndrome, and various apraxias. Most of these deficits are transient and resolve within weeks of surgery. Debate exists as to the extent of callosotomy (partial versus complete) [11, 12], with many practitioners preferring to perform a staged approach—a partial approach first and, if the patient continues to have seizures, a completion of the division [13].

Lobectomy consists of removing the offending cerebral lobe in which the lesion resides. Hemispherectomy involves the removal of the cerebral hemisphere while hemispherotomy involves disconnection of the frontal, parietal, and occipital lobes through a complete corpus callosotomy and mesial temporal resection. Hemispherotomy has in general supplanted both anatomic and functional hemispherectomy for pediatric epilepsy patients, as it minimizes the degree of tissue resection and decreases the degree of intraoperative blood loss, length of hospital stay, need for shunt, and reoperation rate for recurrent seizures [14]. Hemispherotomy and lobectomy both have common surgical risks including bleeding, infection, aseptic meningitis, stroke, hydrocephalus, and possible recurrent seizures requiring further surgery. Mortality has been reported to be 1% with hemispherotomy [15]. 

Stereotactic thermal ablation is a technique of precise destruction of an epileptogenic target by heat. This can be performed by radiofrequency ablation [16] or by delivering the thermal energy by application of a laser into the target [17]. Both heating methods are minimally invasive, and when a laser is used, the operation can be performed in real-time MR Thermography, allowing for maximal target destruction and collateral damage avoidance. 

MST is another palliative option for patients with focal DRE where a lesionectomy or lobectomy cannot be performed, usually due to location of the seizure focus on or adjacent to an eloquent cortical location. The procedure involves performing a craniotomy and horizontally transecting coursing intracortical fibers, while at the same time preserving vertically oriented fibers and adjacent blood vessels. The idea is to disrupt the horizontal travel of synchronized ictal discharges while minimizing the risk of neural deficits from cortical lesioning. In a multicenter meta-analysis of 211 patients who underwent MST, patients with generalized epilepsy who underwent MST alone, without focal resection, 71% experienced a >95% reduction in monthly seizure frequency [18]. Postoperative deficits occurred in 19% and included memory deficits, hemiparesis, and visual field cuts. Motor deficits in particular can recover at seven weeks after MST by exam and at 16 weeks postoperatively by fMRI [19].

In this paper we briefly describe the pediatric epileptiform encephalopathies and review the evidence for the aforementioned surgical procedures in those with DRE.

## 3. Neonate and Infant Syndromes

### 3.1. Early Myoclonic Encephalopathy/Ohtahara Syndrome

EME and Ohtahara syndrome, also known as early infantile epileptic encephalopathy with suppression burst, are the earliest epileptic encephalopathies described by the ILAE report on classification and terminology [2]. Despite being accepted as independent entities, they share several features, including early age of onset, characteristic electrographic features of burst suppression, in which a high voltage bursting pattern is followed by a nearly flat low amplitude signal, and poor prognosis [20]. Indeed, some studies have suggested that rather than being completely separate diseases, they are variants of the same underlying pathology [21]. There are distinguishable elements however [22]. These include the seizure characteristics which in Ohtahara syndrome tend to be tonic spasms, whereas EME presents more with myoclonias or partial seizures. Also, the burst suppression EEG in Ohtahara syndrome occurs during both sleep and awake states, whereas in EME it is usually seen only during sleep states. Ohtahara syndrome also tends to evolve into further epileptic encephalopathic syndromes, including West syndrome and LGS, while EME typically does not. Finally, and most importantly in regard to potential surgical treatments options, Ohtahara syndrome is usually associated with structural brain lesions, whereas EME is usually secondary to genetic or metabolic disorders. 

The association of Ohtahara syndrome with structural lesions allows for possible curative surgical therapies in which the lesion is removed or disconnected. For example, in 1995, Pedespan et al. described the case of a newborn with tonic unilateral seizures which began on the fifth day of life and were refractory to medical therapy with multiple antiepileptic drugs and steroids [23]. An EEG demonstrated the classic burst suppression pattern during both sleep and wake cycles. MRI imaging revealed an area of right frontotemporal cortical thickening, and the epileptogenic focus was further characterized with ECoG and SPECT as localizing to the precentral area. Surgery, which consisted of removal of the precentral area, was performed at 1 month of age, with subsequent relief of symptoms. At one-year followup, the patient had only a single febrile seizure. Komaki et al. had similar results after performing a lesionectomy in a patient with an Ohtahara associated left prefrontal lesion [24]. After surgery, the patient's seizure frequency dropped from several hundred a day to less than two. However, the seizure frequency began to increase two years later, and followup EEG evaluation showed residual epileptogenic focus in the left hemisphere, so a left functional hemispherectomy was performed at age 3, which resulted in complete seizure freedom until last followup two years later [25]. 

These results are typical for patients with Ohtahara syndrome who undergo surgical treatment. Indeed other groups has also demonstrated significant improvement in seizure frequency after surgical intervention, with most patients demonstrating complete seizure freedom or rare seizures. In general, these results can be divided into two patient populations. The first has an identifiable area of cortical dysplasia corresponding to an epileptogenic focus, for which a lesionectomy can be performed [26, 27]. The second less localizing epileptogenic foci and undergo more extensive resection, such as anterior temporal lobectomies [8] or a hemispherectomy variant [27–30]. Both patient populations show significantly improved results not only in diminished seizure frequency, but also in improved postresection development.

In contrast to Ohtahara syndrome, there are limited surgical options for patients with EME. As mentioned above, while Ohtahara syndrome is typically associated with a structural lesion, EME is more affiliated with disorders of metabolism or genetic errors. As such, there is usually no surgical focus for resection. In addition, these children typically present within the first month of life and progress quickly, making palliative surgeries, such as vagal nerve stimulation, less amenable. 

### 3.2. West Syndrome

The encephalopathic syndromes that occur in infancy include Dravet syndrome and West syndrome. West syndrome is a constellation of findings that includes infantile spasms, developmental delay, and a characteristic EEG finding of hypsarrhythmia. Patients typically present within the first year of life and can be a transition from Ohtahara syndrome. The seizures, or spasms, are characterized by generalized axial extension or flexion movements which last for seconds. Hypsarrhythmia is a disorganized pattern of asynchronous, high amplitude slow waves interspersed with frequent multifocal spike-and-sharp wave discharges. Medical treatment, which consists of Vigabatrin, immunoglobulins [31], or ACTH, is often ineffective [32] and for some carefully selected individuals; surgery offers a potential for significant reduction in seizure frequency. 

The favorable aspects of West syndrome for surgical intervention include its association with unilateral and/or focal lesions including tuberous sclerosis [33], Sturge-Weber [34], hemimegalencephaly [35], heterotopias [36], cortical malformations [34], and brain tumors [37]. However, as described by Kramer et al. [38], overt focal clinical or EEG features, such as asymmetric spasms or asymmetric hypsarrhythmia, can exist in the absence of lateralizing radiographic features, and patients with these features can also often be favorable surgical candidates. As such, Asano et al. [39] described a protocol for determining surgical candidacy for patients with West syndrome, which consists of a presurgical evaluation with MRI, PET, and EEG video monitoring on all patients who are nonresponsive to medical treatment with Vigabatrin and ACTH. Candidates for surgery are those in which all studies show concordant lateralization. If the patient is already hemiparetic, then a hemispherectomy/hemispherotomy is also considered. However, if the MRI does not show a lesion or the lesion is in close proximity to the sensory-motor cortex in a nonhemiparetic patient, the surgery proceeds in two stages. The first consists of chronic EEG recording with subdural electrodes over the region of interest followed by the second stage, which entails cortical resection if the epileptogenic foci are identifiable and outside the sensory-motor cortex. 

There have been several studies demonstrating effective reductions in seizure frequency using this approach or variations of it. For example, in a case series involving 23 patients Chugani et al. [40] demonstrated seizure freedom in 65% of patient at 28 months for patients who underwent either a hemispherectomy or a cortical resection after presurgical evaluation with PET imaging revealed them to be good surgical candidates. Another 13% had a decrease in seizure frequency of greater than 90%. Similar results in the treatment of West syndrome were obtained in earlier smaller series by the same group [40, 41] as well as others [42, 43]. 

In patients who are not deemed surgical candidates for resection, usually secondary to discordant lateralization between the EEG, MRI, and PET data, palliative options are available. These include corpus callosotomy and VNS, with the goal of preventing disruptive seizure consequences, including drop attacks or reducing, but not eliminating, seizure frequency. Pinard et al. [44, 45] have shown efficacy in reducing both drop attacks and seizure frequency using callosotomy and Iwasaki et al. [46] have even demonstrated complete remission after this procedure. Vagal nerve stimulation has also shown some promise. Although only two of the 64 patients studied with VNS by Cersósimo et al. [47] had West Syndrome, both patients exhibited a reduction of seizures of 50% or more.

### 3.3. Dravet Syndrome

Dravet syndrome, also known as severe myoclonic epilepsy of infancy, is a rare form of childhood epilepsy that appears in the first year of life. The seizures are characterized as hemiconvulsive or generalized tonic clonic (GTC) and are often precipitated by fever. A mutation in the SCN1A gene, which encodes for a voltage gated sodium channel, has been found in upwards of 80% of patients who present with Dravet syndrome [48]. The prognosis for these patients is poor. From a surgical standpoint, since the disease processes involve diffuse hyperexcitability, resective surgery is not feasible [49].

Palliative surgeries have been tried, including both VNS and DBS, and Zamponi et al. [50] describe the initial results in eight patients with Dravet syndrome who have been treated with VNS. From their study, the mean seizure rate reduction was 12% at three months, 6% at six months, and 31% at a year, with most patients experience some reduction in seizure frequency. Andrade et al. [51] describe the results of two adult patients treated with deep brain stimulation of the anterior nucleus and centromedian nucleus of the thalamus and followed for ten years. The first patient showed significant improvement with an immediate 81% reduction in seizure frequency, and the second showed a gradual decline from 15 to approximately five seizures per month over the course of ten years. Of note is that the site of stimulation was changed from the centromedian nucleus to the anterior nucleus of the thalamus in the second patient. Recently, at the ILAE 2013 conference, CC was presented as a treatment in two patients with Dravet syndrome and SCNI1 abnormality (Ritter et al., unpublished).

### 3.4. Myoclonic Encephalopathy in Nonprogressive Disorders

MSNE is characterized by the recurrence of long lasting myoclonic status epilepticus in infants [52]. While the etiology is often idiopathic, it is often found to be associated with a genetic disorder, typically Angelman syndrome, or fetal anoxic brain injury [52]. Patients present within the first year of life with early onset seizures, frequent myoclonic status, abnormal jerking movements, mental retardation, hypotonia, and a generalized spike-and-wave or delta-theta wave pattern on EEG [52, 53]. The myoclonic movements can often be difficult to detect due to the patients other abnormal movements including absence seizures, which may delay diagnosis. Patients with MSNE typically have a poor prognosis with worsening neuropsychological and motor function over time. The primary management of patients is with AEDs, although there is frequent drug resistance. In the current literature there are no reported cases of surgical intervention for seizure control in those that have MSNE. 

## 4. Childhood Syndromes

### 4.1. Epilepsy with Myoclonic Astatic Seizures

 MAE, or Doose syndrome, is a form of generalized epilepsy that occurs in young children. It is characterized by (1) normal development prior to seizure onset; (2) primarily generalized myoclonic, astatic, or myoclonic-astatic seizures; short absences; or GTC seizures; and (3) a generalized spike-and-wave EEG pattern [54]. Structural abnormalities of the brain are typically absent on imaging. The first line treatment for MAE varies by practitioner, but good results have been reported with a combination of AEDs, including ethosuximide, levetiracetam, valproate or lamotrigine, and the ketogenic diet [55]. Prognosis in MAE patients is variable, but it has been reported that nearly two-thirds of patients may experience seizure freedom following medical therapy [55, 56].

Surgery for MAE is palliative and usually consists of VNS placement. There are no randomized-controlled trials on surgical therapy, with most of the literature consisting of case reports and small case series [47, 57–61]. Seizure reduction with VNS in this patient population is variable, ranging from no reduction [57] to becoming seizure-free at two years [58]. Due to the small number of patients reported it is difficult to make an assessment regarding the utility of VNS for this population. The natural history itself is variable with many children experiencing improvement over time further confounding the effects of VNS.

### 4.2. Lennox-Gastaut Syndrome

 LGS is a childhood epilepsy syndrome that is characterized by multiple seizure types, cognitive dysfunction; a generalized, slow spike-and-wave pattern on EEG (2–2.5 Hz); and tonic fast activity during sleep [62, 63]. Seizure types that may occur include atonic, tonic, myoclonic, or absence seizures. LGS accounts for approximately 4% of all childhood epilepsy, with a prevalence of 0.26 per 1000 live births in the United States [64]. The etiology of LGS can be divided into idiopathic and symptomatic/structural causes. Most cases of LGS are secondary to meningitis/encephalitis, tuberous sclerosis, vascular malformations, hypoxic ischemic injury, or traumatic brain injury. Prognosis is generally poor with significant mental deterioration over time. Medical treatment typically consists of AED polytherapy, immunotherapy, and the ketogenic diet [65]. 

 Surgical options for LGS are based on its etiology. For symptomatic LGS with focal lesions on MRI, resective surgery including lesionectomy, lobectomy, or hemispherectomy/hemispherotomy may be performed. In a series of 27 children and adolescents with LGS by Lee et al. [66], 23 had symptomatic LGS with MRI findings. Of these, 21 underwent lobar or multilobar resection, and 6 underwent hemispherotomy. Overall, both surgical techniques resulted in 74% of the 27 symptomatic LGS patients becoming seizure-free or nearly seizure-free. Hemispherotomy patients had the highest reduction in seizure frequency, followed by multilobar and then lobar surgery patients. 

 For patients with idiopathic LGS, those without identified structural etiologies, palliative surgery using either VNS [59, 67–73], corpus callosotomy [67, 69, 74, 75], or deep brain stimulation [76] has been reported. VNS has been shown to reduce seizure frequency in LGS patients to a variable degree in many studies [10, 21–27]. One of the largest studies, by Frost et al. [72], was a multicenter retrospective study that included 50 LGS patients with a median age of 13 years old who underwent VNS implantation. The median reduction in seizure frequency was 42% at 1 month, 58.2% at 3 months, and 57.9% at 6 months. At 6 months, data were available for only 24 patients, of which 58% had a ≥50% reduction in seizure frequency. Partial seizures responded the poorest out of all seizure types. The most common adverse events that occurred with stimulation included voice alteration or hoarseness in 44% and increased coughing with stimulator setting adjustments in 30%. In another large retrospective study by Helmers et al. [68], 43 LGS patients underwent VNS implantation. Average seizure frequency reduction at 3 months and 6 months was 26.6% and 47.1%, respectively. In addition to hoarseness and voice alteration, other adverse events noted to be unique to the pediatric population with VNS include drooling and hyperactivity [68, 72]. The efficacy of VNS in general tends to increase over time, up to 36 months after implant [77], though recent reports have failed to confirm this observation [78].

 Corpus callosotomy (CC) is another palliative technique used in refractory LGS patients, albeit a more invasive one. Several patient series have been reported for CC in LGS patients [25, 27–29]. One of the largest series is by Cukiert et al. [73] in which LGS patients with either VNS or CC were compared. Twenty patients were in the VNS group, 24 patients in the CC group. All patients were considered to have idiopathic LGS or LGS-like disease. At two years, 10% of the VNS group were seizure-free, versus 0% in the CC group. There were 10% and 16% nonresponders in the VNS and CC groups, respectively. Both groups experienced significant improvement in quality of life and attention, likely due to an overall reduction in mean seizure frequency in each group. There were distinct differences in terms of effectiveness for different seizure types. Callosotomy worked better in terms of mean frequency reduction of atonic seizures while VNS was more effective in terms of myoclonic seizures. Reduction in seizure frequency following CC has been previously shown to be associated with improved quality of life, despite no improvement in mental performance [79]. A meta-analysis of VNS and CC studies for LGS patients by Lancman et al. [69] confirmed that CC was more effective for atonic seizures versus VNS (70% versus 26.3% responder rate), but no significant difference was found for other seizure types including myoclonic, tonic, GTC, and complex-partial seizures. There was a 3.7% VNS complication rate versus a 8.3% complication rate in CC. The analysis was limited by small CC sample size overall, reliability of seizure reporting, and incomplete data in many studies. On small series of patients by Jalilian et al. [74] attempted to address the question of whether partial division of the corpus callosum (anterior 2/3) is as effective in seizure reduction for LGS patients. They found that out of eight LGS patients there was no significant difference between the two techniques. Given the potential for significant seizure reduction from a partial division, we favor a staged approach, first performing a partial CC and completing it if necessary.

 DBS for intractable LGS patients is not common in practice but has been reported in the literature. Velasco et al. [76] reported a series of 13 LGS patients with GTC and atypical absence seizures. Bilateral electrodes were implanted in the centromedian nucleus. Stimulation resulted in an 80% overall seizure reduction, with 12 patients experiencing >50% reduction in mean seizure frequency at 18 months and two patients seizure-free. Two patients had explantation of hardware due to skin erosion over hardware. While not standard of care, the interest in DBS for epilepsy has been increasing as of late [80] and may be a viable treatment option for DRE patients in the near future.

### 4.3. Epileptic Encephalopathy with Continuous Spike and Wave during Sleep Including Landau-Kleffner Syndrome

CSWS is an epileptiform disorder in children characterized by various seizure types (although no tonic seizures), with a sleep induced EEG pattern characterized by a subclinical spike-and-wave pattern that occurs continuously during slow wave sleep. Patients may experience neuropsychological and/or motor deterioration over time. Seizures can occur both while being asleep and awake. Landau-Kleffner syndrome is considered to be on the same spectrum of epilepsy as CSWS [2, 81] and usually presents with progressive loss of receptive and expressive language ability with associated EEG disturbances. Both CSWS and Landau-Kleffner present in otherwise healthy children, and both carry a relatively good prognosis, though many children retain permanent neurological deficits [82, 83]. Patients with CSWS and unilateral polymicrogyria may have a better seizure prognosis than patients with bilateral polymicrogyria and CSWS [84]. 

First line treatment for patients with CSWS is pharmacologic, but surgery is an option for DRE patients. Lesionectomy/lobectomy, CC, and hemispherotomy have all been described for CSWS [85–87]. In one of the larger reported series, Peltola et al. [86] describe 13 patients with CSWS between 3 and 10 years old. Two patients underwent resection of a focal lesion, one underwent hemispherectomy, and eight patients underwent a CC. Of the lesionectomy/lobectomy group, two of three became free of disabling seizures, versus none in the CC group. Cognitive decline was halted in all but one patient. Two of the CC group patients had a significant reduction in disabling seizures. None of the patients with significant seizure reduction had the characteristic CSWS EEG findings postoperatively. Similar results for patients with CSWS were seen in other small series [85, 87]. In patients with CSWS associated with unilateral polymicrogyria and DRE, some would argue for watchful waiting given the good prognosis with these patients over time. However, surgery in this case may be the more conservative option, given the relatively low risk of CC, to help prevent further seizures and associated cognitive decline.

MST has been reported as a treatment option for patients with Landau-Kleffner syndrome [88–92]. Most patients undergoing this procedure have significant improvement in language function, although of those that do improve, many do not return to an age appropriate level [88–92]. In a recent small series 70% showed language improvement, but none returned to normal function, and 50% had reduction in seizure frequency [91]. Although the natural history of Landau-Kleffner syndrome is that of improvement in neurological function over time, surgery should be considered a viable option to maximize that neurologic improvement.

### 4.4. Epileptic Encephalopathy in Localization-Related Epilepsy

The conditions reviewed above serve well to illustrate the condition of epileptic encephalopathy, but these conditions are rare. The most commonly encountered epileptic encephalopathy in an epilepsy treatment center is that which presents in untreated or undertreated localization-related epilepsy or focal epilepsy. This can occur in Rasmussen's disease, Sturge-Weber syndrome, tuberous sclerosis, hypothalamic hamartoma, or epilepsy related to postnatal stroke, malformations of cortical development (MCD) (focal cortical dysplasia, hemimegalencephaly, polymicrogyria, and microdysgenesis), and posttraumatic, tumor-related, or postinfectious epilepsy. High-resolution MRI is an essential part of the seizure workup in all epileptic encephalopathy patients to identify these structural abnormalities, all of which may be addressed surgically. The clinical scenario typically involves young age and high seizure burden [93, 94]. Frequently, multiple antiepileptic medications are employed in high doses, making the distinction between epileptic encephalopathy and medication effect difficult. Despite this confusion, however, improvement or resolution of the encephalopathy is frequently encountered with seizure cessation alone.

In most series improvement or resolution of epileptic encephalopathy is reflected in improved cognition and behavior, either formally tested or subjectively reported by the patient's family [95]. Cognitive and behavioral improvements after hemispherectomy have been noted since the onset of the application of the technique to epilepsy [96, 97], even being credited for the resolution of Von Monakow's diaschisis [98]. Duration, etiology, and hemispheric localization seem to influence cognitive outcome [99]. ESES [86, 87, 100], Rasmussen's, and other acquired epileptic etiologies [101] had favorable cognitive outcome in contrast to vascular injury, malformations of cortical development, and hemimegalencephaly. Laterality also influenced cognitive outcome, with right, nondominant hemisphere surgery patients showing more cognitive improvement that left [102], dominant hemispheric patient or patients with contralateral hemispheric anatomic abnormalities [99]. Boshuisen et al. showed that contralateral MRI abnormalities negated cognitive improvement after hemispherectomy (38% versus 0% improvement without and with contralateral MRI abnormalities, resp.) but that contralateral EEG findings did not [88, 99]. In an interesting case report of a 22-year-old patient with Sturge-Weber that underwent left hemispherectomy at age 3 years, testing showed adequate language function similar to IQ matched controls but significantly impaired visual spatial skills [103]. Age of onset and duration of epilepsy determined cognitive outcome in some studies [101]. Villarejo-Ortega et al. showed that although patients with perinatal vascular lesions had less cognitive improvement after-hemispherectomy than late onset acquired epilepsy such as Rasmussen's encephalopathy, there was an inverse linear correlation between the degree of cognitive improvement and the age at surgery [104]. Lettori et al. also suggested that young age at surgery affords better cognitive improvement [105]. The percentage of patients enjoying developmental improvement varies from 57% to 72% [87, 106–108]. The degree of presurgical intellectual disability influenced postsurgical cognitive improvement, with the most severely intellectually disabled enjoying less cognitive improvement than less affected children [106]. The duration of this developmental improvement after epilepsy surgery has also been shown to extend up to three years postoperatively [109]. The patients with the greatest cognitive improvement have CSWS [87].

Although developmental improvement can be an attainable goal, the majority of encephalopathic children undergoing epilepsy surgery experience intellectual stability in contrast to their presurgical decline [105, 110–113]. Devlin et al. published their hemispherectomy experience on 33 children that showed lack of cognitive decline generally and improvement in 4 patients across all categories of pathology, developmental, acquired, or progressive [110]. In examining the poor outcome etiology of hemimegalencephaly, Battaglia et al. reported that better cognitive outcomes were predicted by good presurgical cognition, less radiologic malformation, and good functional and anatomic integrity of the contralateral hemisphere [114]. However, the findings of cognitive improvement are not apparent in all series, with Korkman et al. showing a lack of cognitive improvement in the Finnish series and no impact of presurgical IQ, age, sex, type of surgery, or age at surgery on outcome [115]. Pulsifer et al. also showed minimal cognitive improvement in the Johns Hopkins series of hemispherectomy by hemidecortication, assigning the underlying pathology as the main predictor of cognitive outcome [116]. The same center published similar findings in analyzing the cognitive outcomes of Sturge Weber syndrome patients undergoing hemispherectomy [117]. Lastly, a lack of neuropsychological improvement after hemispherectomy for stroke-related epilepsy was also reported by Scavarda et al. [118].

Special consideration is needed when contemplating hemispheric surgery on the dominant side in the developing brain. Consultation concerning outcome is partially dependent upon the estimation of language development or improvement. Although there is conflicting literature [102, 119–122] on the topic, a review of 43 hemispherectomized children revealed that the “earlier the better” hypothesis of Kennard [123] is not generally supported when considering language [124, 125]. This review concluded that the etiology is the most predictive factor in language outcome after hemispherectomy and that age of onset, age at surgery, side of resection, and post-op seizure control were only influential when etiology was taken into account [125]. For instance, age of onset and age at surgery were only predictive of recovery in acquired, right hemispheric surgery and not in developmental etiologies or in left hemisphere surgery. Additionally, when it was predictive, it was opposite to the Kennard hypothesis in that the older children recovered better language [125]. The side of surgery was not predictive within their whole study group, but in acquired disease, operating the nondominant hemisphere clearly had better language outcomes [125]. Lastly, postoperative seizure control only mattered as it relates to language development in the developmental etiology group [125]. Case studies [120, 126] and fMRI data [121] support the role of the right hemisphere in language support and development. Short-term verbal memory and verbal intelligence also positively predicted composite language in hemispherectomy patients in another series [102].

Another clinical scenario where epileptic encephalopathy is encountered is in the treatment of hypothalamic hamartoma [127]. These lesions present with gelastic, or laughing, seizures in the infant or even neonatal period that is frequently mistaken for normal behavior. The seizures typically progress to a refractory epilepsy with developmental deterioration and behavior changes such as rage attacks [128]. With the hamartoma proven to be the seizure onset zone [129], effective treatment has been focused upon the hypothalamic lesion itself. There have been many operations designed to resect or destroy the hamartoma, with a range of morbidity. Frontal or temporal resections targeting foci of secondary epileptogenesis are invariably ineffectual [130]. The open transcallosal, interforniceal resection [131] has been shown to be effective in 57% cases but also associated with high rate of morbidity, with 50% hypothalamic obesity, 45% short-term memory deficit, and 15% permanent diabetes insipidus [132, 133]. This high morbidity drove the development of ablative approaches such as stereotactic radiofrequency thermoablation [134], stereotactic I-125 interstitial radiotherapy [135], and stereotactic radiosurgery [136] with efficacy averaging from 37% to 50% but with reduced morbidity. 

Endoscopic resection [137] or disconnection [138] was developed to reduce the morbidity of the approach (memory deficit) but still was hampered by hypothalamic consequences such as DI and obesity. Most recently, MR-guided stereotactic laser interstitial therapy (MRgLITT) has been applied to the treatment of hypothalamic hamartomas with low morbidity [17].

## 5. Conclusion

The evidence for surgical intervention in patients with pediatric encephalopathy that is drug resistant is sparse. There is no class I evidence supporting surgical intervention in this patient population. Based on the limited available evidence, patients with focal epileptic foci or structural lesions causing their encephalopathic syndrome may be considered for either lesionectomy/lobectomy, MST, or hemispherotomy as a treatment option. We recommend high-resolution MRI in all patients with an epileptiform encephalopathy to identify potential epileptic lesions. If no obvious structural abnormality on imaging or EEG, a palliative procedure such as VNS or CC may be an option. DBS for pediatric intractable epilepsy, while promising, should be considered investigational at this time, until further evidence becomes available.
